# Endophytic Colonisation of *Solanum lycopersicum* and *Phaseolus vulgaris* by Fungal Endophytes Promotes Seedlings Growth and Hampers the Reproductive Traits, Development, and Survival of the Greenhouse Whitefly, *Trialeurodes vaporariorum*

**DOI:** 10.3389/fpls.2021.771534

**Published:** 2021-11-19

**Authors:** Vongai M. Paradza, Fathiya M. Khamis, Abdullahi A. Yusuf, Sevgan Subramanian, Sunday Ekesi, Komivi S. Akutse

**Affiliations:** ^1^International Centre of Insect Physiology and Ecology (icipe), Nairobi, Kenya; ^2^Department of Zoology and Entomology, University of Pretoria, Hatfield, South Africa; ^3^Forestry and Agricultural Biotechnology Institute (FABI), University of Pretoria, Hatfield, South Africa

**Keywords:** *Trialeurodes vaporariorum*, endophytes, life-history parameters, progeny survival, colonisation persistence, systemic resistance, biopesticides

## Abstract

In the scope of mitigating the negative impacts of pesticide use and managing greenhouse whiteflies, *Trialeurodes vaporariorum* sustainably, 16 endophytic fungal isolates from five different genera (*Beauveria*, *Trichoderma*, *Hypocrea*, *Bionectria*, and *Fusarium*) were screened for their ability to colonise two preferred host plant species, namely, tomato (*Solanum lycopersicum* L.) and French bean (*Phaseolus vulgaris* L.), through seed inoculation. Seven and nine isolates were endophytic to *P. vulgaris* and *S. lycopersicum*, respectively, where significant differences in the endophytic colonisation rates were observed among the fungal isolates in *P. vulgaris* and its plant parts, with a significant interaction between the isolates and plant parts in *S. lycopersicum*. *Hypocrea lixii* F3ST1, *Trichoderma asperellum* M2RT4, *Trichoderma atroviride* F5S21, and *T. harzianum* KF2R41 successfully colonised all the plant parts of both hosts and therefore were selected and further evaluated for their endophytic persistence, effect on plant growth, and pathogenicity to *T*. *vaporariorum* adults and F1 progeny. The four endophytes remained in both host plants for the 5-week assessment with varied colonisation rates related to the strong interaction with the time, isolates, and plant parts in both hosts. The effect of the same endophytes on the different host growth parameters varied in *P. vulgaris* and *S. lycopersicum*, with *T. asperellum* M2RT4 not boosting the growth in both host plants while *T*. *atroviride* F5S21 resulted in enhanced shoot biomass in *S. lycopersicum*. *T. atroviride* F5S21 and *T*. *harzianum* KF2R41 inoculated *S. lycopersicum* plants and *H. lixii* F3ST1, *T. asperellum* M2RT4, and *T*. *harzianum* KF2R41 inoculated *P. vulgaris* plants had significantly lower oviposition, while nymph development in both hosts was significantly prolonged in all the endophytically–colonised plants. The endophytes *H. lixii* F3ST1 and *T. asperellum* M2RT4 significantly reduced the longevity/survival of the exposed *T. vaporariorum* adults and the progeny in both *S. lycopersicum* and *P. vulgaris*. The findings demonstrate the attributes of the various endophytes in host plant growth promotion as well as their effects on the life-history parameters of *T. vaporariorum* and could consequently be developed as potential endophytic fungal-based biopesticides for the sustainable management of the pest in *S. lycopersicum* and *P. vulgaris* cropping systems.

## Introduction

The greenhouse whitefly, *Trialeurodes vaporariorum* Westwood (Hemiptera: Aleyrodidae) is a globally serious pest in vegetable and ornamental crop production systems, attacking crops within the families Crucifareae, Legumimoseae, Malvaceae, Solanaceae, and Asteraceae (Kim et al., 2014; Wang et al., 2017). The damage from *T. vaporariorum* is caused by both the adults and the nymphs through phloem-feeding, resulting in the loss of nutrients and subsequent low plant productivity (Arnó i Pujol et al., 2009; Gao et al., 2017). Both the adults and nymphs have a sap-sucking feeding behaviour and use a specialised stylet that passes through the epidermis and mesophyll layers allowing them to feed exclusively from the phloem bundle (Wang et al., 2017). During feeding, whiteflies also excrete honeydew, which is a substrate for sooty mould (*Capnodium* sp.). The sooty mould reduces the photosynthetic capacity which directly affects the growth and productivity of the plant. It also reduces the aesthetic value of the crop, and this is important in crops like ornamentals, leafy vegetables, and fruits because their marketability depends on their appearance. In addition to their direct infestation to the crops, they also cause indirect important economic damage through their transmission of several plant viruses. *T. vaporariorum* is a vector of several criniviruses such as Tomato chlorosis virus and Tomato infectious chlorosis virus, and torradoviruses like Tomato torrado virus, although some of these viruses can also be transmitted by other whitefly species, such as *T. abutiloneus* (Haldeman) and *Bemisia tabaci* (Gennadius) (Hemiptera: Aleyrodidae) (Navas-Castillo et al., 2011). Losses from *T. vaporariorum* transmitted plant viruses depend on the nature of the virus, the crop, and its developmental stage, as well as the disease incidence (Lapidot et al., 2014). For example, losses resulting from Lettuce infectious yellows virus resulted in a yield reduction ranging from 50 to 75% in lettuce and 20 to 30% in sugar beets (Wisler et al., 1998), while strawberry yield losses ranged between 15 and 20% caused by Strawberry pallidosis associated virus (Wintermantel, 2004). In addition, losses due to Tomato infectious chlorosis virus amounted to US$ 2 million in tomatoes (Wisler et al., 1998; Wintermantel, 2004).

The proliferation of greenhouses in Kenya has provided optimum climatic conditions for the high reproduction rates and proliferation of *T. vaporariorum* (Jones, 2003; Wainaina et al., 2018). From the greenhouses, the pest subsequently escapes into open fields, hence, its presence in both open and protected agricultural systems (Lapidot et al., 2014; Perring et al., 2018). French bean, *Phaseolus vulgaris* L., and tomato, *Solanum lycopersicum* L. are two of the most important vegetables in the horticultural sector of Kenya. French bean is an important export vegetable (Okello and Swinton, 2007; Gogo et al., 2014b) which is exported as fresh or canned, contributing to around 21.3% of the total value of vegetable exports, second to mixed vegetables (Horticultural Crops Directorate, 2019). In terms of production and value, tomato is the second leading vegetable after potato, contributing up to 20.1% of the total value of vegetables (Horticultural Crops Directorate, 2019) and mainly grown for the domestic market (Minot and Ngigi, 2004; Mithöfer et al., 2008; Gogo et al., 2014a). Since the cultivation of these crops by smallholder farmers in Kenya is mainly carried out in open fields, it makes them highly predisposed to whiteflies infestations. Together with other pests such as leafminer (*Liriomyza* spp.), thrips (*Frankliniella* spp.), red spider mites (*Tetranychus* spp.), and aphids (*Aphis* spp.), whiteflies are also listed among the major pests of tomatoes and French beans in Kenya (Nyasani et al., 2012; Gogo et al., 2014a). Tomatoes also serve as a propagative host for some of the criniviruses vectored by the pest (Wintermantel, 2004).

Farmers rely heavily on synthetic pesticides to control whiteflies (Nderitu et al., 1997) and this has been the basis to suppress the pest populations (Kim et al., 2014; Lapidot et al., 2014). However, factors such as high fecundity rates, a short life cycle which enables quick population build-up within a short time, a waxy cuticle layer that limits penetration by contact pesticides, and polyphagy which ensures abundant alternative hosts have led to the low success of the chemical control (Hirano et al., 1993; Gilbertson et al., 2011; Abd-Rabou and Simmons, 2012). This has thus placed them among the few pest species that have driven intensive pesticide use. The intensive application of pesticides, especially in areas of high infestations, as is often the case with *T. vaporariorum* (Palumbo et al., 2001) and in high-value crops like vegetables where pest thresholds should remain low, has led to the development of pesticide-resistant whitefly populations (Denholm et al., 1998). Therefore, whiteflies are now reported as major vegetable pests with high resistance to all classes of pesticides (Capinera, 2001; De Bon et al., 2014; Coffey et al., 2015), and with resurgence often seen even after spraying (Legg et al., 2014). Because of the negative impacts that high pesticide use poses to the environment, biodiversity, and public health, the adoption of sustainable crop protection methods has increasingly become key to sustainable agriculture.

The management of *T. vaporariorum* especially in protected agriculture has been successful using biological control agents such as parasitoids *Encarsia formosa* (Gahan) and *Eretmocerus eremicus* (Rose and Zolnerowich) (Hymenoptera: Aphelinidae) (Gonzalez et al., 2016); predators like *Amblyseius swirskii* (Athias-Henriot) (Acari: Phytoseiidae) and *Delphastus catalinae* (Horn) (Coleoptera: Coccinellidae) (Spence et al., 2020), and some species of entomopathogenic fungi, namely, *Beauveria bassiana* (Balsamo) Vuillemin, *Metarhizium anisopliae* (Metschnikoff) Sorokin (Hypocreales: Clavicipitaceae), and *Isaria fumosorosea* (Wize) Brown and Smith (Hypocreales: Cordycipitaceae) (Gökçe et al., 2005; Gonzalez et al., 2016).

Some entomopathogenic fungi, known as endophytes, live symbiotically within plant tissues for part of or their entire life cycle and are known to protect plants against abiotic and biotic stressors (Lacey et al., 2015; Vidal and Jaber, 2015; Jaber and Ownley, 2018). Endophytes stimulate the synthesis of secondary metabolites such as terpenoids, phenols, and phytoalexins volatile oils which confer resistance to several insects, resulting in the deterrence of feeding, oviposition, stem boring, sap sucking, and leaf mining by pests (Gao et al., 2011; Kambrekar, 2016; Agbessenou et al., 2020). The systemic effect of endophytes against insects has been shown in several other insects including diamondback moth [*Plutella xylostella* (L.)] (Lepidoptera: Plutellidae) (Batta, 2013; Sun et al., 2018), Silverleaf whitefly (*B. tabaci*) (Gennadius) (Hemiptera: Aleyrodidae) (Garrido-Jurado et al., 2017), pea leafminer (*Liriomyza huidobrensis*) (Blanchard) (Diptera: Agromyzidae) (Akutse et al., 2013), tomato leafminer (*Tuta absoluta*) (Meyrick) (Lepidoptera: Gelechiidae) (Klieber and Reineke, 2016; Agbessenou et al., 2020), cotton aphid (*Aphis gossypii*) (Glover) (Homoptera: Aphididae) (Lopez et al., 2014), mealybugs (*Planococcus ficus*) (Signoret) (Hemiptera: Pseudococcidae) (Rondot and Reineke, 2018), and spider mites (*Tetranychus urticae*) (Koch) (Acari: Tetranychidae) (Pappas et al., 2018). Another additional benefit from some endophytes is their potential to promote plant growth (Mayerhofer et al., 2013; Hassan, 2017; Bamisile et al., 2018a,b, 2020; Russo et al., 2019). Therefore, the study of plant–endophyte interactions is an important approach in continuing to build the knowledge on endophytes as candidates for the development of biopesticides against insects like *T. vaporariorum*, which have become resistant to synthetic pesticides. The objectives of the study were to assess the endophytic colonisation and persistence of some selected fungal isolates in *P. vulgaris*. and *S. lycopersicum*, evaluate their effects on plant growth, and assess the systemic effects of the endophytically colonised host seedlings on the development and survival of *T. vaporariorum* adults and progeny.

## Materials and Methods

### Experimental Site, Design, and Parameters Measured

The experiments were conducted in screen houses and the Arthropod Pathology Unit laboratories at the International Centre of Insect Physiology and Ecology (*icipe*), Duduville Campus, Nairobi – Kenya (1.2219°S, 36.8967°E). Sixteen fungal isolates were screened for colonisation and systemic induction assessment in *S. lycopersicum* and *P. vulgaris*. The four best performing isolates, based on their ability to colonise the root, stem, and leaf tissue of both host plants, were selected to assess their endophytic persistence, effect on plant growth parameters, fecundity, development, and survival of *T. vaporariorum* adults and first-generation (F1) progeny.

### Insect Rearing

Whitefly populations were initially collected from eggplants (*Solanum melongena* L.) grown in the greenhouses at *icipe*’s Duduville Campus. Two colonies were reared separately in screen houses on potted tomatoes (*S. lycopersicum* L., cv. Moneymaker) and French beans (*P. vulgaris* L., cv Goal) in Plexiglas cages (40 cm × 60 cm × 80 cm) (Millenium Chuma Limited, Kenya) with fine muslin walls for more than four generations before use in experiments (Kakimoto et al., 2007; Jaber et al., 2018). The whiteflies were identified as *T. vaporariorum* through the PCR amplification of the mitochondrial 16S ribosomal RNA (rRNA) gene fragment using the WF-F (5′-CGCCTGTTTAACAAAAACAT-3′) and WF-R (5′-CCGGTCTGAACTCAGATCACGT-3′) primers (Frohlich et al., 1999; Alhudaib et al., 2014). The sequencing of the PCR products obtained from 10 whiteflies confirmed the identity of the species under study. The sequences have been deposited in the National Center for Biotechnology Information (NCBI) GenBank database under accession numbers OK500114, OK500115, OK500116, OK500117, OK500118, and OK500119. The colonies were maintained inside screen houses with natural light conditions at 25 ± 2°C, 65% relative humidity, and a photoperiod of 12:12 h light/dark. Whitefly adults ≤5 days old were used for all bioassays (Pakkianathan et al., 2015), taking into account the approximate preoviposition period of whiteflies, ranging between 1.4 and 3.6 days (Sharaf and Batta, 1985; Salas and Mendoza, 1995; Capinera, 2001).

### Fungal Culture and Viability Assessment

The first experiment was the screening of 16 fungal isolates from five different genera; 8 *B. bassiana –* ICIPE 273, 281, 284, 609, 621, 676 (isolated from the soil), ICIPE 279 (from coleopteran larvae), and ICIPE 35 (from coffee berry borer); 1 *Hypocrea lixii –* F3ST1 (from maize); 4 *Trichoderma* – *Trichoderma* spp. F2LT4, and *T. asperellum* M2RT4 (from monocots), *T. harzianum* KF2R41, and *Trichoderma atroviride* F5S21 (from onion); 2 *Bionectria ochroleuca* – F3R21 and F3S21 (from onion); and 1 *Fusarium proliferatum –* NF2S51 (from onion). The isolates were obtained from the *icipe* Arthropod Germplasm Centre for subculture. All the isolates were cultured on a Potato Dextrose Agar (PDA) (OXOID CM0139, Oxoid Ltd., Basingstoke, United Kingdom) and incubated in darkness at 25 ± 2°C for 14–21 days. Conidia were harvested by scraping off the agar surface into 10 ml sterile distilled water with 0.05% Triton X-100 (MERCK KGaA, Darmstadt, Germany) in a universal bottle containing glass beads. The resulting suspension was vortexed to get a uniform suspension and the spore concentration was adjusted to 1 × 10^8^ conidia/ml using a Neubauer haemocytometer (VWR International, United States) (Inglis et al., 2012).

The conidial viability was assessed before each bioassay under a microscope by inoculating 0.1 ml of the 3 × 10^6^ conidia/ml suspension onto four fresh plates of PDA for each isolate using a glass spreader. The Petri dishes were incubated in complete darkness for 18 h at 25 ± 2°C. The percentage germination was calculated by counting the number of germinated conidia per hundred randomly selected conidia in a selected field covered by four coverslips under a microscope at 400× magnification (Leica DM500). Conidia with visible germ tubes of about twice the diameter of the conidium were scored as viable.

### Seed Inoculation With Fungal Isolates

Prior to inoculation, the *S. lycopersicum* and *P. vulgaris* seeds were surface sterilised in 70% ethanol for 2 min, followed by 1.5% sodium hypochlorite solution for 3 min with constant shaking, rinsed with three washes in sterile distilled water, and dried aseptically (Akutse et al., 2013). To check the effectiveness of the surface sterilisation procedure, tissue imprinting and plating of the last rinse water were conducted on a PDA media (Inglis et al., 2012). The absence of fungal growth after incubation was indicative of the effectiveness of the sterilisation procedure. The *S. lycopersicum* and *P. vulgaris* seeds were soaked in 1 × 10^8^ conidia/ml fungal suspensions for 18 and 2 h, respectively (Akutse et al., 2013). The control seeds were soaked in sterile 0.05% Triton X–100 solution. Field soil mixed with manure at a ratio of 5:1 autoclaved at 121°C for 2 h and left to cool for 72 h prior to sowing was used as the planting substrate. Five seeds were planted per pot (8 cm diameter and 7 cm height), and later thinned to three after germination. The plants were grown in screen houses for 3 weeks at 25 ± 2°C under natural light conditions with no additional fertiliser. Watering was done as necessary to keep adequate soil moisture for the growth of the seedlings.

### Colonisation Assessment

Three-week-old seedlings were uprooted and washed with tap water to remove the soil. For each treatment, a total of 12 plants were used. The plants were divided into three parts (root, stem, and leaves), cut into 1 cm root and stem pieces and 1 mm^2^ leaf pieces, and were surface sterilised under a laminar flow hood (Jaber et al., 2018). Five plant pieces per replicate were then randomly selected for each plant part and were surface sterilised as described earlier above. The pieces were plated equidistant from each other on a PDA supplemented with antibiotics (0.25 g/L w/v chloramphenicol) (Akello et al., 2007; Batta, 2013). The Petri dishes were incubated at 25°C for 14 days to assess the fungal growth from within the plant tissues. The proportion of the plant parts colonised by the inoculated fungal isolate was calculated for each treatment as the number of plant pieces showing fungal outgrowth divided by the total number of plant pieces plated. The evaluation was based on the morphological characteristics of the inoculated fungus that colonised the incubated plant part, and only the colonisation by the inoculated fungi was scored as positive. Slides prepared from the mother plates were used for comparison in morphological identification (Dash et al., 2018). The treatments were arranged in a completely randomised design and replicated four times over time.

### Endophytic Persistence and Evaluation of Seedling Growth Parameters

The four isolates *H. lixii* F3ST1, *T. asperellum* M2RT4, *T. atroviride* F5S21, and *T. harzianum* KF2R41 that successfully colonised both host plants were selected for the subsequent experiments. To examine the endophytic persistence and the effect of the isolates on the plant growth parameters, seed inoculation with the above isolates and controls was done as described in the colonisation experiment, and the plants were grown singly in pots (14 cm diameter, 14 cm height) under a completely randomised block design. Eight replicate plants per treatment were destructively sampled each week for 5 weeks starting at 1-week post-germination. After recording the growth parameters, the plants were uprooted for the colonisation experiment. The growth parameters that were evaluated were the plant height (base of the stem to its tip), number of fully developed leaves, leaf width (widest part of the leaf lamina), and leaf length (distance from the leaf apex to its stalk). The fresh and dry shoot weights were also measured only in the final week to assess the total accumulated shoot biomass for the entire growing period. The dry shoot weight was measured by cutting off 2 cm above the base of the pseudostem, and drying the shoots in a hot air oven at 60°C for 48 h (Akello et al., 2007; Sun et al., 2018).

### Bioassays on Survival, Fecundity, and Nymph Development

Forty newly emerged adults of *T. vaporariorum* (20 males and 20 females) were exposed to 3-week old endophytically colonised plants inside Plexiglas cages (30 cm × 30 cm × 30 cm) for 48 h for the oviposition bioassay (Greenberg et al., 2000). Each cage had a single plant that constituted a treatment and replicated four times. After the exposure time, the insects were blown off the leaves, and the number of eggs was counted under a dissecting microscope (×35; Leica EZ4 HD).

To evaluate the nymph development, the inoculated and endophytically colonised 3-week-old plants were placed with the infested plants for 48 h for oviposition and then removed. The position of 40 settled first instar nymphs was marked by placing a small black dot near each nymph using a fine-tipped permanent marker. All the developmental stages were followed on the same marked nymphs. The number of nymphs that had developed into second and fourth instar was counted at 11–13 and 20 days post-exposure, respectively (Mascarin et al., 2013; Malekan et al., 2015), using a guide on nymph sizes by Naranjo and Ellsworth (2017). The adult emergence was determined by counting the number of adults that had emerged from the pupal cases 10 days after the onset of emergence.

The survival of adult *T. vaporariorum* on inoculated plants was assessed by exposing 3-day-old whiteflies (100 flies at a ratio of 1:1 male: female) to 3-week endophytically colonised plants in Plexiglas cages (30 cm × 30 cm × 30 cm) for 48 h. The cages were maintained in the screen house at 25 ± 2°C, 65% relative humidity, and a photoperiod of 12:12 h light/dark. Survival was monitored by counting the number of dead/surviving insects daily for 15 days for the whitefly adults exposed to endophyte inoculated plants and 25 days for the progeny emerging from inoculated plants. All the dead whiteflies were surface sterilised with 1% sodium hypochlorite solution followed by three rinses using sterile distilled water and placed in Petri dishes lined with a moist filter paper for the mycosis test. The control insects were exposed to endophyte-free plants in all three experiments.

### Statistical Analyses

The proportional data (root, stem, and leaf pieces colonised by the various fungal isolates and nymph development data showing second instar, fourth instar, and adult emergence counts) were analysed using logistic regression in the generalised linear model (GLM) with binomial distribution and logit link function. The significantly different means were identified by Tukey’s honestly significant difference (Tukey’s HSD) considering a significance level of 5% (Mascarin et al., 2013). The isolates which did not colonise any plant part and the control plant data were not included in the analysis (Greenfield et al., 2016). The survival analysis based on the Kaplan–Meier product-limit method was used to determine the survival probability functions of the adults and progeny exposed to different fungal treatments and controls. The survival function curves for different fungal treatments and the controls were compared using the log-rank test (Agbessenou et al., 2020). The plant height, leaf length, leaf width, and shoot weight were analysed using ANOVA, and the differences in means were separated using the Student–Newman–Keuls (SNK) test. All the data sets were previously checked for the homogeneity of variances and normality among the treatments using the Bartlett (Snedecor and Cochran, 1989) and Shapiro–Wilk tests (Shapiro and Wilk, 1965), respectively. The number of eggs and leaves was modelled as a Poisson distribution, taking into account the dispersion (Akello et al., 2007). All statistical analyses were performed using the R Statistical package version R-3. 5. 2 (R Core Team, 2018).

## Results

### Endophytic Colonisation of *S. lycopersicum* and *P. vulgaris* by Fungal Isolates

**FIGURE 1:**
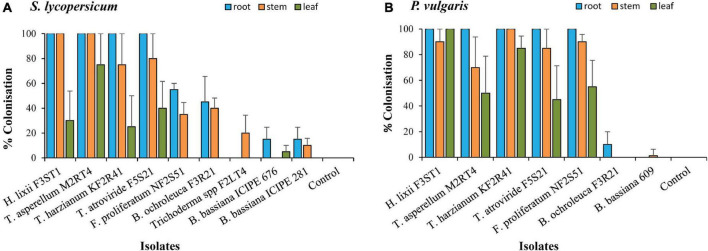
The colonisation of different parts of *Solanum lycopersicum* **(A)** and *Phaseolus vulgaris* **(B)** plants by endophytic fungal isolates of *Beauveria bassiana* (ICIPE 609, 676, and 281), *Bionectria ochroleuca* F3R21, *Fusarium proliferatum* NF2S51, *Hypocrea lixii* F3ST1, *Trichoderma* spp F2LT4, *T. asperellum* M2RT4, *T. atroviride* F5S21, and *T. harzianum* KF2R41. Error bars represent the SE (±SE) of the means at 95% CI (Tukey’s HSD test, *P* ≤ 0.05).

The conidial viability for all the isolates was >90%. From the screening experiment, only the results for the isolates that colonised at least one plant part are presented. Nine isolates were able to colonise *S. lycopersicum* compared with seven for *P. vulgaris* (Figures 1A,B). The colonisation rates differed across the isolates (χ^2^ = 300.00, *df* = 6, *P* < 00001) and plant parts (χ^2^ = 55.4, *df* = 2, *P* < 0.0001) for *P. vulgaris*, while those in *S. lycopersicum* showed a significant interaction between the isolates and plant parts (χ^2^ = 34.90, *df* = 16, *P* < 0.01). The colonisation rates highly depended on the fungal isolate, plant parts, and the host; for example, *B. ochroleuca* F3R21 managed to colonise 45% (roots) and 40% (stem) in *S. lycopersicum*, while in *P. vulgaris*, the root colonisation was only 10% with no stem colonisation. *B. bassiana* isolates ICIPE 676, ICIPE 609, and ICIPE 281 generally had the lowest colonisation rates in both hosts compared with the other isolates from other genera. *H. lixii* F3ST1, *T. asperellum* M2RT4, *T. atroviride* F5S21, and *T. harzianum* KF2R41 recorded 100% root colonisation in both host plants, the stem colonisation ranged between 75–100 and 45–100% in *S. lycopersicum* and *P. vulgaris*, respectively, while the leaf colonisation rates were 25–75% in *S. lycopersicum* and 45–100% in *P. vulgaris*. *F. proliferatum* NF2S51 managed to fully colonise all the plant parts in *P. vulgaris* with the rates of 100% (roots), 90% (stem), and 55% (leaf) (Figure 1A), while in *S. lycopersicum*, the root and stem colonisation rates were 55 and 35%, respectively. However, unlike in *P. vulgaris* (Figure 1B), *F. proliferatum* NF2S51 failed to colonise *S. lycopersicum* leaves (Figure 1A). No fungal growth was observed in the control plants, tissue imprinted, and plated last rinse water.

#### Endophytic Colonisation Persistence for Selected Isolates

**FIGURE 2:**
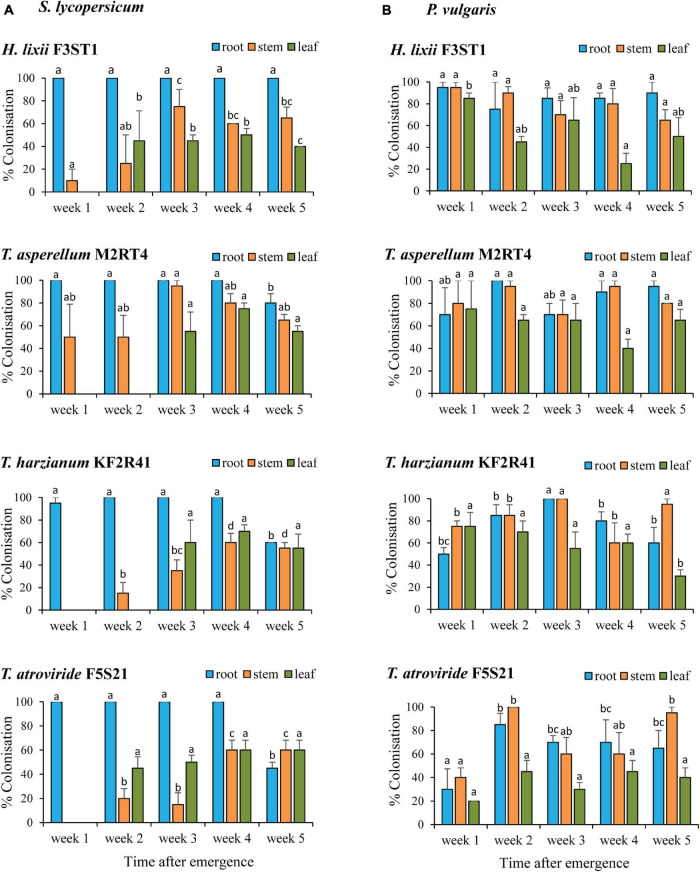
Endophytic persistence of *H. lixii* F3ST1, *T. asperellum* M2RT4, *T. atroviride* F5S21, and *T. harzianum* KF2R41 in host plants *S. lycopersicum* **(A)** and *P. vulgaris* **(B)** 5 weeks post-germination. Error bars represent the SE (±SE) of the means at 95% CI (Tukey’s HSD test, *P* ≤ 0.05). Bars indicated by the same letters for the same plant part (root, stem, leaf) across weeks are not significantly different.

*Hypocrea lixii* F3ST1, *T. asperellum* M2RT4, *T. atroviride* F5S21, and *T. harzianum* KF2R41 were selected to assess the persistence of the colonisation within the hosts based on the above screening results which showed their ability to colonise all the plant parts of both host plants. These isolates were able to remain endophytic for the entire 5-week evaluation period, although the colonisation rates were dependent on the isolate, plant parts, and time (Figure 2).

In *S. lycopersicum*, there were significant interactions between the isolates and plant parts (χ^2^ = 44.03, *df* = 6, *P* < 0.0001) and between the time and plant parts (χ^2^ = 148.29, *df* = 8, *P* < 0.0001). During the first week, only the roots were colonised by *T. atroviride* F5S21 and *T. harzianum* KF2R41 while *T. asperellum* M2RT4 and *H. lixii* F3ST1 managed to colonise both the roots and stems. By the second week, *H. lixii* F3ST1 and *T. atroviride* F5S21 achieved full colonisation of all the plant parts, whereas, during the same period, *T. asperellum* M2RT4 and *T. harzianum* KF2R41 had colonised only the roots and stems, and managed to fully colonise the entire host plant from the 3rd-week post-inoculation (Figure 2A).

The endophytic colonisation persistence pattern in *P. vulgaris* was different, with all the endophytic fungal isolates achieving full colonisation from the first week and remaining almost constant throughout the 5 weeks for most of the isolates and plant parts (Figure 2B). There was a significant interaction between the isolates, plant parts, and time (χ^2^ = 60. 71, *df* = 24, *P* < 0.0001) with regards to the colonisation rates. For example, *T. atroviride* F5S21 had lower colonisation rates of 30, 40, and 20% in the root, stem, and leaf tissue, respectively, during the first week compared with the other three isolates which had colonisation rates ranging from 50 to 95% (root), 75 to 95% (stem), and 75 to 85% (leaf) (Figure 2B). Leaf colonisation by *T. harzianum* KF2R41 was lower in the final week (week five) compared with the first 4 weeks of evaluation.

#### Effect of Endophytes on *S. lycopersicum* and *P. vulgaris* Growth Parameters

The evaluation of the growth parameters in *S. lycopersicum* showed that the endophytes did not enhance the various growth parameters in the weekly assessments compared with the control treatment. The number of leaves recorded for the *T. asperellum* M2RT4 inoculated plants was significantly lower than in the *H. lixii* F3ST1, *T. atroviride* F5S21, and control treatments in the third and fourth week but showed a significant gain in the fifth week. In other parameters, the *T. asperellum* M2RT4 inoculated plants showed a significantly lower plant height and leaf length than all the other treatments starting from the third and fourth week, respectively, until the fifth week (Table 1). Significant differences concerning leaf width amongst the treatments were also recorded in the first and fourth weeks. The *H. lixii* F3ST1 inoculated plants exhibited lower leaf width growth compared with the *T. atroviride* F5S21 inoculated plants in the first week. In the fourth week, the *T. asperellum* M2RT4 inoculated plants showed a lower leaf width growth than the *T. atroviride* F5S21 inoculated and control plants. However, at week five, all treatments recorded similar leaf width with no significant difference (Table 1).

**TABLE 1:** The effects of the seed inoculation of *Hypocrea lixii* F3ST1, *Trichoderma asperellum* M2RT4, *T. atroviride* F5S21, and *T. harzianum* KF2R41 on the growth parameters of *Solanum lycopersicum.*

Parameter	Week 1	Week 2	Week 3	Week 4	Week 5
**Leaf number**					
*H. lixii* F3ST1	4.00 ± 0 a	4.62 ± 0.18 ab	6.87 ± 0.29 b	9.25 ± 0.36 b	10.25 ± 0.25 ab
*T. atroviride* F5S21	4.00 ± 0 a	5.25 ± 0.25 b	7.25 ± 0.31 b	9.37 ± 0.32 b	11.25 ± 0.72 b
*T. asperellum* M2RT4	4.00 ± 0 a	4.50 ± 0.18 a	5.87 ± 0.22 a	8.25 ± 0.16 a	9.50 ± 0.18 a
*T. harzianum* KF2R41	4.00 ± 0 a	4.75 ± 0.16 ab	6.75 ± 0.25 ab	8.62 ± 0.18 ab	9.87 ± 0.22 ab
Control	4.00 ± 0 a	5.12 ± 0.12 ab	7.12 ± 0.12 b	9.37 ± 0.18 b	10.62 ± 0.18 ab
χ*2*	0	0.68	1.41	0.94	1.41
df	4,35	4,35	4,35	4,35	4,35
P	1	0.017	0.001	0.002	0.008
**Plant height (cm)**					
*H. lixii* F3ST1	4.33 ± 0.35 a	16.42 ± 1.17 a	19.31 ± 0.96 a	31.24 ± 1.79 a	47.35 ± 2.32 a
*T. atroviride* F5S21	4.33 ± 0.17 a	16.7 ± 0.81 a	20.21 ± 1.25 a	32.56 ± 1.93 a	49.43 ± 2.80 a
*T. asperellum* M2RT4	4.28 ± 0.31 a	13.58 ± 0.86 a	15.5 ± 1.00 b	24.10 ± 1.87 b	39.51 ± 3.47 b
*T. harzianum* KF2R41	4.58 ± 0.14 a	15.35 ± 0.65 a	19.82 ± 0.25 a	31.05 ± 1.25 a	47.97 ± 2.39 a
Control	4.45 ± 0.16 a	16.87 ± 1.30 a	19.33 ± 0.89 a	33.34 ± 2.02 a	51.42 ± 2.95 a
F	0.23	1.89	3.43	4.21	2.59
df	4,35	4,35	4,35	4,35	4,35
P	0.91	0.133	0.018	0.006	0.05
**Leaf length (cm)**					
*H. lixii* F3ST1	2.87 ± 0.24 a	6.75 ± 0.33 a	7.97 ± 0.45 a	15.48 ± 0.72 a	17.54 ± 0.71 a
*T. atroviride* F5S21	3.25 ± 0.24 a	6.96 ± 0.34 a	8.38 ± 0.67 a	16.66 ± 1.21 a	19.20 ± 1.22 a
*T. asperellum* M2RT4	2.60 ± 0.24 a	6.20 ± 0.34 a	7.30 ± 0.42 a	12.08 ± 0.76 b	14.69 ± 0.76 b
*T. harzianum* KF2R41	2.78 ± 0.20 a	7.31 ± 0.30 a	9.05 ± 0.61 a	16.00 ± 0.75 a	17.94 ± 0.49 a
Control	3.42 ± 0.12 a	7.23 ± 0.34 a	8.88 ± 0.48 a	17.12 ± 0.76 a	18.46 ± 0.85 a
F	2.41	2.23	1.73	5.32	4.15
df	4,35	4,35	4,35	4,35	4,35
P	0.06	0.155	0.164	0.001	0.007
**Leaf width (cm)**					
*H. lixii* F3ST1	0.86 ± 0.08 b	6.75 ± 0.29 a	9.83 ± 0.61 a	15.04 ± 0.80 ab	18.15 ± 0.72 a
*T. atroviride* F5S21	1.22 ± 0.08 a	7.77 ± 0.51 a	10.12 ± 0.99 a	15.86 ± 1.08 a	19.21 ± 1.25 a
*T. asperellum* M2RT4	0.96 ± 0.07 ab	6.45 ± 0.40 a	8.41 ± 0.61 a	11.88 ± 0.79 b	15.61 ± 0.96 a
*T. harzianum* KF2R41	0.95 ± 0.10 ab	7.31 ± 0.46 a	10.57 ± 0.61 a	15.15 ± 1.10 ab	18.39 ± 0.89 a
Control	1.11 ± 0.05 ab	7.71 ± 0.18 a	11.12 ± 0.51 a	16.85 ± 1.02 a	19.45 ± 1.22 a
F	3.04	2.23	2.17	3.68	2.17
df	4,35	4,35	4,35	4,35	4,35
P	0.029	0.085	0.09	0.013	0.09

*The SE is shown on the mean (±SE).*

*Means followed by the same letter in a column are not significantly different (SNK test, P ≤ 0.05).*

The assessment of the key growth parameters in *P. vulgaris* showed no significant differences in the number of leaves among the treatments in all the weeks of evaluation. However, significant differences in plant height, leaf length, and leaf width were recorded only in the first week. The *T. asperellum* M2RT4 inoculated and control plants exhibited lower plant height and leaf width concerning the other treatments (Table 2). The plants inoculated with *T. atroviride* F5S21 showed significantly greater leaf length growth than the *T. asperellum* M2RT4 inoculated and control plants but did not differ significantly from the other treatments. From the second week to the final week of evaluation (week five), no significant differences were observed for all the growth parameters in all the treatments (Table 2).

**TABLE 2:** The effect of the seed inoculation of *H. lixii* F3ST1, *T. asperellum* M2RT4, *T. atroviride* F5S21, and *T. harzianum* KF2R41 on the growth parameters of *Phaseolus vulgaris.*

Parameter	Week 1	Week 2	Week 3	Week 4	Week 5
**Leaf number**					
*H. lixii* F3ST1	2.00 ± 0 a	3.87 ± 0.12 a	5.00 ± 0.32 a	8.12 ± 0.87 a	9.62 ± 0.94 a
*T. atroviride* F5S21	2.00 ± 0 a	3.87 ± 0.12 a	5.37 ± 0.32 a	9.00 ± 0.88 a	11.75 ± 0.79 a
*T. asperellum* M2RT4	2.00 ± 0 a	4.00 ± 0 a	5.50 ± 0.56 a	9.12 ± 0.63 a	12.50 ± 0.86 a
*T. harzianum* KF2R41	2.00 ± 0 a	3.75 ± 0.16 a	5.37 ± 0.16 a	8.87 ± 0.74 a	12.25 ± 1.16 a
Control	2.00 ± 0 a	4.00 ± 0 a	5.37 ± 0.32 a	9.00 ± 0.42 a	12.37 ± 0.76 a
χ*2*	0	0.09	0.21	0.59	4.07
df	4,35	4,35	4,35	4,35	4,35
P	1	0.448	0.929	0.877	0.09
**Plant height (cm)**					
*H. lixii* F3ST1	8.47 ± 0.56 a	13.82 ± 2.10 a	24.53 ± 2.85 a	34.96 ± 3.73 a	37.92 ± 3.71 a
*T. atroviride* F5S21	8.20 ± 0.45 a	14.41 ± 1.56 a	27.03 ± 2.77 a	37.43 ± 4.06 a	40.90 ± 3.97 a
*T. asperellum* M2RT4	6.16 ± 0.30 b	13.37 ± 1.02 a	25.35 ± 2.08 a	34.60 ± 3.02 a	37.28 ± 3.21 a
*T. harzianum* KF2R41	8.12 ± 0.53 a	13.52 ± 0.93 a	24.21 ± 2.60 a	35.72 ± 4.23 a	41.61 ± 3.21 a
Control	5.57 ± 0.48 b	15.42 ± 1.61 a	27.83 ± 2.23 a	38.97 ± 3.48 a	42.57 ± 2.99 a
F	7.77	0.3	0.39	0.24	0.45
df	4,35	4,35	4,35	4,35	4,35
P	0.0001	0.872	0.814	0.913	0.766
**Leaf length (cm)**					
*H. lixii* F3ST1	4.68 ± 0.23 abc	8.57 ± 0.91 a	12.86 ± 1.03 a	13.83 ± 1.19 a	16.66 ± 1.42 a
*T. atroviride* F5S21	5.21 ± 0.06 a	10.76 ± 0.87 a	13.76 ± 0.86 a	14.26 ± 0.80 a	17.18 ± 0.45 a
*T. asperellum* M2RT4	4.08 ± 0.15 c	9.47 ± 0.61 a	12.00 ± 0.85 a	12.81 ± 0.85 a	17.52 ± 0.88 a
*T. harzianum* KF2R41	4.95 ± 0.24 ab	8.95 ± 1.04 a	13.28 ± 0.64 a	14.55 ± 0.56 a	17.52 ± 0.54 a
Control	4.40 ± 0.17 bc	9.88 ± 0.50 a	13.83 ± 0.45 a	14.92 ± 0.52 a	17.88 ± 0.41 a
F	5.63	1.09	0.89	0.97	0.3
df	4,35	4,35	4,35	4,35	4,35
P	0.001	0.374	0.476	0.435	0.872
**Leaf width (cm)**					
*H. lixii* F3ST1	3.62 ± 0.17 a	12.80 ± 1.43 a	19.42 ± 1.85 a	19.90 ± 1.97 a	24.72 ± 2.22 a
*T. atroviride* F5S21	3.76 ± 0.10 a	14.91 ± 1.19 a	18.36 ± 1.12 a	20.81 ± 1.12 a	25.80 ± 0.97 a
*T. asperellum* M2RT4	2.90 ± 0.09 b	13.43 ± 0.66 a	16.73 ± 1.03 a	19.43 ± 1.12 a	25.68 ± 1.78 a
*T. harzianum* KF2R41	3.46 ± 0.09 a	13.43 ± 1.42 a	19.05 ± 0.82 a	20.36 ± 0.88 a	24.73 ± 0.76 a
Control	3.00 ± 0.13 b	14.8 ± 1.07 a	19.81 ± 1.10 a	20.53 ± 0.61 a	26.25 ± 0.82 a
F	8.18	0.6	0.95	0.19	0.22
df	4,35	4,35	4,35	4,35	4,35
P	0.001	0.658	0.446	0.939	0.923

*The SE is shown on the mean (±SE).*

*Means followed by the same letter in a column are not significantly different (SNK test, P ≤ 0.05).*

The endophytic inoculation of *S. lycopersicum* by *T. atroviride* F5S21 resulted in a significant increase in the cumulative plant shoot biomass at the end of the evaluation period (week 5), for both the fresh shoot weight (*F* = 6.95, *df* = 4, 35, *P* < 0.001) and dry shoot weight (*F* = 6.92, *df* = 4, 35, *P* < 0.001). The *S. lycopersicum* plants endophytically colonised by *T. atroviride* F5S21 gained 13.94 and 14.36% more fresh and dry shoot weight, respectively, when compared with the control, while *T. asperellum* M2RT4 had a 53.83% lower dry shoot weight than the control (Table 3). The comparison of the two endophytes showed that *T. asperellum* M2RT4 had 66.90% lower dry shoot weight than *T. atroviride* F5S21. No significant differences among the treatments were recorded for both the fresh and dry shoot weight in *P. vulgaris* (Table 3).

**TABLE 3:** The effect of seed inoculation of *H. lixii* F3ST1, *T. asperellum* M2RT4, *T. atroviride* F5S21, and *T. harzianum* KF2R41 on the plant shoot biomass at 5 weeks post-inoculation.

	*Solanum lycopersicum*		*Phaseolus vulgaris*	
	Fresh shoot weight (g)	Dry shoot weight (g)	Fresh shoot weight (g)	Dry shoot weight (g)
*H. lixii* F3ST1	39.61 ± 2.76 ab	3.14 ± 0.25 ab	22.07 ± 4.55 a	3.60 ± 0.74 a
*T. atroviride* F5S21	48.42 ± 3.15 a	3.73 ± 0.31 a	26.70 ± 3.63 a	4.09 ± 0.55 a
*T. asperellum* M2RT4	26.81 ± 2.92 c	1.86 ± 0.22 c	27.36 ± 3.01 a	4.25 ± 0.46 a
*T. harzianum* KF2R41	35.02 ± 2.75 bc	2.65 ± 0.24 b	26.18 ± 3.79 a	3.75 ± 0.54 a
Control	42.11 ± 3.59 ab	3.23 ± 0.30 ab	31.07 ± 1.33 a	4.67 ± 0.23 a
F	6.95	6.92	0.87	0.63
df	4,35	4,35	4,35	4,35
P	0.0002	0.0003	0.49	0.638

*The SE is shown on the mean (±SE). Means followed by the same letter in a column are not significantly different (SNK test, P ≤ 0.05).*

### Effect of Endophytically Colonised *S. lycopersicum* and *P. vulgaris* Host Plants on *T. vaporariorum* Oviposition and Nymphal Development

**FIGURE 3:**
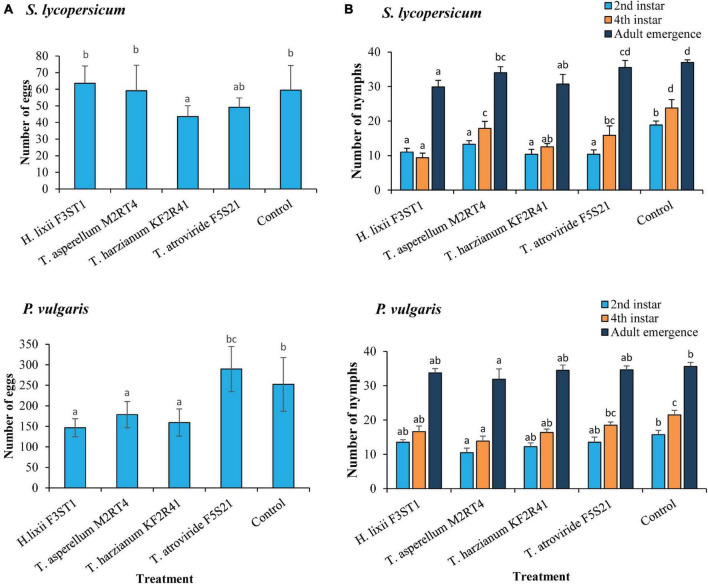
*Trialeurodes vaporariorum* oviposition **(A)** and nymph development **(B)** negatively affected by endophytically colonised *S. lycopersicum* and *P. vulgaris* plants inoculated with *H. lixii* F3ST1, *T. asperellum* M2RT4, *T. atroviride* F5S21, and *T. harzianum* KF2R41. Error bars represent the SE (±SE) of the means at 95% CI (Tukey’s HSD test, *P* ≤ 0.05). Bars indicated by the same letters are not significantly different.

The endophytes had a significant effect on *T. vaporariorum* oviposition in both the *S. lycopersicum* (χ^2^ = 41.52, *df* = 4, *P* < 0.0001) and *P. vulgaris* (χ^2^ = 593.11, *df* = 4, *P* < 0.0001) host plants (Figure 3A). However, there was high variability between the two endophytically colonised host plants for the same isolates with regards to the reproduction traits. For example, the oviposition on the *S. lycopersicum* endophytically colonised by *H. lixii* F3ST1 (63.62 ± 10.40 eggs) and *T. asperellum* M2RT4 (59.12 ± 15.38 eggs) was not significantly different from the control (59.5 ± 14.78 eggs), but the same isolates had significantly lower egg numbers on the endophytically colonised *P. vulgaris* plants by *H. lixii* F3ST1 (146.5 ± 21.88 eggs) and *T. asperellum* M2RT4 (178.62 ± 31.95 eggs) as compared with the control (252.37 ± 65.39 eggs) (Figure 3A). The isolate which consistently recorded the lowest oviposition in both host plants was *T. harzianum* KF2R41 (Figure 3A). In general, the number of eggs laid on *S. lycopersicum* across all treatments (43.5 ± 6.53–63.62 ± 10.4 eggs) was significantly lower than those laid on *P. vulgaris* (146.5 ± 21.88–289.5 ± 54.90 eggs) (Figure 3A).

The effect of the endophytes was also evident in the various stages of nymphal development in both host plants. On the endophytically colonised *S. lycopersicum* plants, a significant effect was observed regarding the number of nymphs that developed into the second instar (χ^2^ = 46.32, *df* = 4, *P* < 0.0001), with all the four endophytes equally suppressing nymphal development, with nymph numbers ranging from (10.37 ± 1.42–13.25 ± 1.03 nymphs) as compared with the control (18.87 ± 1.12 nymphs). Similarly, the effect of the treatments on the number of fourth instar nymphs was also significant (χ^2^ = 101.34, *df* = 4, *P* < 0.0001) (Figure 3B). The isolate which had the greatest negative effect on the development of nymphs to the fourth instar was *H. lixii* F3ST1 (23.4 ± 3.37% nymphs), followed by *T. harzianum* KF2R41 (31.2 ± 2.45% nymphs), *T. atroviride* F5S21 (39.6 ± 6.77% nymphs), and *T. asperellum* M2RT4 (44.6 ± 5.14% nymphs), while the control had the highest number of fourth instar nymphs (59.3 ± 6.11% nymphs). For adult emergence, there were also significant differences among the treatments (χ^2^ = 55.64, *df* = 4, *P* < 0.0001), with the lowest number of insects emerging from the *H. lixii* F3ST1 endophytically colonised plants (74.6 ± 4.78% insects), followed by *T. harzianum* KF2R41 (76.8 ± 6.93% insects), *T. asperellum* M2RT4 (85.0 ± 4.35% insects), *T. atroviride* F5S21 (88.7 ± 5.15% insects), and the control (92.5 ± 1.82% insects) (Figure 3B).

The evaluation of the nymphal development in *P. vulgaris* also showed significant differences at each of the different stages of *T. vaporariorum* development, second instar (χ^2^ = 13.72, *df* = 4, *P* = 0.0108), fourth instar (χ^2^ = 26.18, *df* = 4, *P* < 0.0001), and adult emergence (χ^2^ = 12.08, *df* = 4, *P* = 0.0516). Generally, the endophyte which greatly suppressed nymphal development was *T. asperellum* M2RT4 (26.2 ± 3.25%; 34.7 ± 3.52% nymphs and 79.7 ± 7.60% insects at second, fourth instar, and adult emergence, respectively) followed by the other three isolates *T. harzianum* KF2R41, *T. atroviride* F5S21, and *H. lixii* F3ST1 as compared with the control (39.3 ± 3.12% nymphs at second instar, 53.7 ± 3.35% nymphs at fourth instar and 89.0 ± 2.90% emerged insects) (Figure 3B).

### Systemic Effects of Endophytically Colonised *S. lycopersicum* and *P. vulgaris* on the Survival of *T. vaporariorum* Adults

**FIGURE 4:**
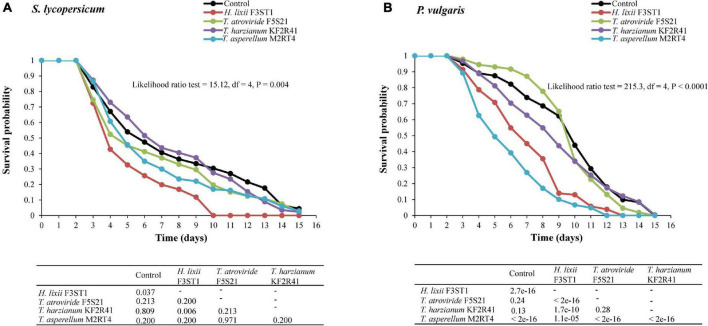
Survival curves of *T. vaporariorum* adults after exposure to 3-week-old endophytically colonised *S. lycopersicum* **(A)** and *P. vulgaris* **(B)** plants by different fungal isolates of *H. lixii* F3ST1, *T. asperellum* M2RT4, *T. atroviride* F5S21, and *T. harzianum* KF2R41.

There was a significant treatment effect on the survival of adult *T. vaporariorum* that were exposed to the endophytically colonised *S. lycopersicum* (proximate log-rank test = 15.12, *df* = 4, *P* = 0.004) and *P. vulgaris* (proximate log rank test = 215.3, *df* = 4, *P* < 0.0001) (Figures 4A,B). For the inoculated *S. lycopersicum* plants, *H. lixii* F3ST1 outperformed all the other isolates and significantly reduced the survival of the exposed insects compared with the control (*P* = 0.037) (Figure 4A). At five days post-exposure, the survival in the *H. lixii* F3ST1 inoculated plants was 32.70% compared with the control which was at 53.95%. By the 10th day, the mortality in the *H. lixii* F3ST1 treatment had reached 100% (no survival) while the control insects exhibited greater longevity with a survival probability of 30.44%, and median times to death of 4 (4–5) and 6 (5–8) days, respectively (Figure 4A).

In the *P. vulgaris* inoculated plants, *H. lixii* F3ST1 (*P* < 0.0001) and *T. asperellum* M2RT4 (*P* < 0.0001) significantly reduced *T. vaporariorum* survival compared with the control (Figure 4B). Five and ten days post-exposure, the survival rates in the different treatments were 49.37 and 6.65% for *T. asperellum* M2RT4; and 70.79 and 12.99% for *H. lixii* F3ST1 as compared with 87.50 and 44.01% for the control. All the insects were dead at days 12 and 13 post-exposure for *T. asperellum* M2RT4 and *H. lixii* F3ST1, respectively, while the insects in the control group achieved 100% mortality at day 15. The median times to death were 10 (10–11) days (control), 7 (6–8) days (*H. lixii* F3ST1), and 5 (5–6) days (*T. asperellum* M2RT4) (Figure 4B). No mycosis was observed on the insect cadavers.

### Systemic Effects of Endophytically Colonised *S. lycopersicum* and *P. vulgaris* on the First Generation Progeny of *T. vaporariourum*

**FIGURE 5:**
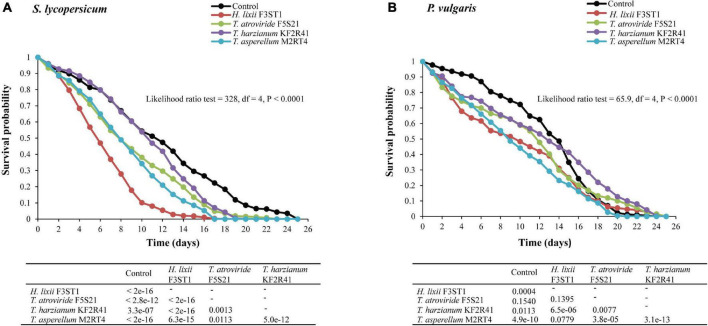
Survival curves of *T. vaporariorum* first generation (F1) progeny after emergence from endophytically colonised *S. lycopersicum* **(A)** and *P. vulgaris* **(B)** plants by different fungal isolates of *H. lixii* F3ST1, *T. asperellum* M2RT4, *T. atroviride* F5S21, and *T. harzianum* KF2R41.

There were significant differences among the treatments in the survival rates of the first generation progeny which emerged from the endophytically colonised *S. lycopersicum* (proximate log-rank test = 328, *df* = 4, *P* < 0.0001) and *P. vulgaris* (proximate log-rank test = 65.9, *df* = 4, *P* < 0.0001) (Figure 5). In *S. lycopersicum*, the survival rates at 5 and 10 days post-exposure and their respective median time to death for the different potent endophytes were 56.78 and 10.08% with 6 (6–7) days for *H. lixii* F3ST1, 73.91 and 34.21% with 8 (8–9) days for *T. asperellum* M2RT4, 71.05 and 38.13% with 8 (7–9) days for *T. atroviride* F5S21, and 84.52 and 53.92% with 11 (10–12) days for *T. harzianum* KF2R41, compared with 81.38 and 54.34% with 12 (10–13) days for the control, respectively (Figure 5A).

A similar comparison with *P. vulgaris* showed that the survival rates of the F1 progeny at 5 and 10 days post-exposure and their respective median time to death for the different endophytic isolates were 63.66 and 48.31% with 10 (8–12) days for *H. lixii* F3ST1, 71.78 and 44.16% with 9 (9–10) days for *T. asperellum* M2RT4, 71.5 and 58.9% with 12 (12–13) days for *T. atroviride* F5S21, and 76.83 and 59.22% with 13 (12–14) days for *T. harzianum* KF2R41, compared with 90.63 and 72.28% with 14 (13–15) days for the control, respectively (Figure 5B).

## Discussion

Endophytes play an important role in protecting plants against different pest species, pathogens, and environmental stresses (Mayerhofer et al., 2013; Gao et al., 2017; Gange et al., 2019). The success of this important association, however, depends on the compatibility between the host plant species and the endophyte. Compatibility determines whether the endophytes fail to establish or is able to proliferate within the different plant tissues. The results from the current study showed the successful colonisation of two plant species, *S. lycopersicum* and *P. vulgaris*, by endophytes from different genera and also demonstrated the influence of the host plant species, endophyte, plant part, and persistence time as key determinants of colonisation efficiency. This was exhibited by the pattern of colonisation which was different for the two host plants. Whereas in *P. vulgaris*, the full endophytic colonisation of all plant parts was achieved from the first week post-inoculation by all the isolates, the colonisation of the entire host plant in *S. lycopersicum* by all the endophytes occurred at the third-week post-inoculation. The colonisation rates within the successive host plant organs are an indication of the time an endophyte requires to passively move through interconnecting tissues to colonise the entire plant (Wagner and Lewis, 2000). The duration has been shown to be dependent on both the host and the endophyte species/strains (Qayyum et al., 2015). Our results showed a migration direction/speed of the endophyte inoculum from the root to the leaves, which was found to be faster in *P. vulgaris* than in *S. lycopersicum.* This observation concurs with what was highlighted by Akello et al. (2007) and Gange et al. (2019) that the differences in physiology and chemistry of different plant species influence colonisation efficiency. Wagner and Lewis (2000) also pointed out how the succulent nature of the corn pith proved ideal for the movement of *B. bassiana* as an endophyte. Since the same endophytes produced different effects in the hosts, it shows that the responses might be host-dependent. In addition, different plant species and different plant parts usually harbour diverse endophytes (Patil et al., 2016; Hassan, 2017), and therefore, successful colonisation also depends on the ability of the artificially inoculated endophyte to outcompete the other endophytes already present in the plant (Gao et al., 2011; Kambrekar, 2016). The host plant may also trigger a response that may be defensive, resulting in variations in the colonisation patterns between the two plant species and plant parts. As the plant ages, changes in the physiology, hormonal composition, and competition of the plant in the utilisation of nutrients with other endophytes might also result in lower colonisation (Gaiero et al., 2013; Russo et al., 2019). This was demonstrated in the *S. lycopersicum* endophytically colonised by *T. atroviride* F5S21, *T. asperellum* M2RT4, and *T. harzianum* KF2R41, which showed a significant decline in the root colonisation levels by the pre–inoculated endophytes in the final week, while a higher diversity of the naturally occurring endophytes was observed during reisolation, which were previously absent. A clearer trend in the decline of colonisation might probably have been observed if the assessment had gone beyond 5 weeks. Further studies are therefore warranted to elucidate these variations in both tomato and French bean plants.

There was very low colonisation by *B. bassiana* isolates in both hosts, 15% (ICIPE 676 and 281) for the roots in *S. lycopersicum* and 1.25% (ICIPE 609) in the *P. vulgaris* stems, which seems consistent with some studies that reported that *B. bassiana* is more competent as an above-ground endophyte than a root coloniser (Meyling et al., 2011; Behie et al., 2015) because of tissue specificity (Compant et al., 2016), with foliar sprays achieving higher colonisation than soil drenching (Donga et al., 2018; Saragih, 2019; Wei et al., 2020). However, since other methods of inoculation by *B. bassiana* such as *P. vulgaris* and *Vicia faba* seed inoculation (Akutse et al., 2013), *S. lycopersicum* root dip (Qayyum et al., 2015), *P. vulgaris* foliar sprays (Afandhi et al., 2019), soil drenching in *Musa* spp (Akello et al., 2007), and stem injection in *Coffea* spp (Posada et al., 2007) resulted in successful high colonisation rates in other studies, the low colonisation in the current study could have been due to host-endophyte incompatibility.

The weekly measurements of various growth parameters did not show significant growth promotions in the inoculated plants in some traits compared with the uninoculated controls. However, the effect of *T. atroviride* F5S21 in enhancing growth in *S. lycopersicum* was evident in the shoot biomass accumulated during the entire growing period. *Trichoderma atroviride* F5S21 gave the highest fresh and dry shoot weight compared with other treatments. Endophytes have been shown to enhance growth in other different plant species such as soya bean (Russo et al., 2019), banana (Akello et al., 2007), turfgrass (Gaggìa et al., 2013), red chilli (Saragih, 2019), neem (Verma et al., 2011), broad bean (Jaber and Enkerli, 2016), and cucumber (Khan et al., 2012). The increase of growth by endophytes is attributed to their role in stimulating the production of plant growth hormones such as auxins, indole acetic acid, and gibberellic acid which are all important for host growth regulation (Kambrekar, 2016; Hassan, 2017; Saragih, 2019). For instance, indole acetic acid is an important compound that integrates the symbiotic relationship between the host plant and the endophyte (Hassan, 2017). However, for *T. asperellum* M2RT4, it was observed that as the plants achieved more colonisation in the different parts, a lower plant height and leaf length were recorded compared with the other treatments in some weeks. The reason for this response could be explained by the fact that since the symbiotic relationship between the host plant and the endophyte is such that it derives its nutrients from the photosynthates of the plants and in turn provides the plant with nitrogen and phosphates (Behie et al., 2017), the increased nutrient demand and carbon drain may be the reason for the corresponding reduction in the speed of growth (Mack and Rudgers, 2008; Rodriguez et al., 2009). Furthermore, the plant also uses an abundant amount of energy to sustain the symbiotic relationship between itself and the endophyte, and therefore, slower growth, in this case, can be viewed as a compensatory effect (Wei et al., 2020). In contrast to *S. lycopersicum*, endophyte inoculation did not affect the *P. vulgaris* shoot biomass while the plant height and leaf width were generally lower for the *T. asperellum* M2RT4 inoculated and control plants only in the first week with no significant differences for the remaining weeks for these growth parameters. As highlighted by Mayerhofer et al. (2013), the contrasts in the results for the growth parameters in which different hosts respond differently to the same endophyte can be attributed to host specificity and may not be reproducible for all hosts. Other examples where endophyte inoculation did not result in enhanced plant growth are cacao (Hanada et al., 2010), tomato (Wei et al., 2020), radish (Sun et al., 2018), and sorghum (Tefera and Vidal, 2009).

The systemic effects of the endophytes on *T. vaporariorum* were also investigated in terms of oviposition, nymphal development, and adult and progeny survival. There were significant variations with the oviposition counts (number of eggs laid) among the different treatments. For instance, the oviposition on the *S. lycopersicum* inoculated with *H. lixii* F3ST1 and *T. asperellum* M2RT4 was the same as the control plants in contrast to *P. vulgaris* where the same treatments had oviposition counts significantly lower than the controls. Despite the high oviposition counts, the same endophytes, *H. lixii* F3ST1 and *T. asperellum* M2RT4, generally managed to suppress nymphal development and adult emergence on the endophytically colonised *S. lycopersicum*. The reason for the difference in the effect of endophytes on the oviposition and nymph development in the current study might possibly be a result of the endophyte-mediated oviposition preferences through volatile cues (Jallow et al., 2008). Feeding on inoculated plants, on the contrary, directly exposes the insect to secondary metabolites such as terpenoids, isoflavonoids, and isocoumarins that have toxic effects which inhibit insect performance (Gaggìa et al., 2013; Jaber and Ownley, 2018; Wei et al., 2020). Similar trends were also observed in other studies in which *Helicoverpa armigera* (Hübner) (Lepidoptera: Crambidae) moths (Jallow et al., 2008) and *T. vaporariorum* (Vidal, 1996) showed oviposition preference on tomato plants inoculated with the *Acremonium strictum* and laid more eggs compared with endophyte–free plants although the *H. armigera* larvae and *T. vaporariorum* nymphs which later developed on the inoculated plants had prolonged development time and a higher mortality rate. Gange et al. (2019) analysed several studies on plant-endophyte-insect interactions and showed that some parameters do not necessarily respond in the same pattern as also observed in our study for oviposition, nymphal development, and adult emergence. Similar studies have also shown the negative systemic effects of endophytes on different life parameters of several insects. *H. lixii* F3ST1 caused the lower pupation, emergence, and survival of *L. huidobrensis* in *Vicia faba* (Akutse et al., 2013), reduced the oviposition and mining activity of *T. absoluta* in both *S. scabrum* and *S. lycopersicum* (Agbessenou et al., 2020), and reduced the feeding and oviposition of *Thrips tabaci* (Lindeman) (Thysanoptera: Thripidae) in *Allium cepa* (Muvea et al., 2014). *Trichoderma asperellum* M2RT4 is also reported to have a negative effect on the development and reproduction of *Acyrthosiphon pisum* (Harris) (Homoptera: Aphididae) in *V. faba* (Akello and Sikora, 2012), while *S. lycopersicum* inoculated with *B. bassiana* caused the lower oviposition and feeding by *B. tabaci* (Wei et al., 2020). In this study, the dead insects which were exposed to the endophytically colonised plants did not show any mycosis. Even though the intercellular presence of endophytic fungi through artificial inoculation/colonisation and their systemic effects on herbivorous pests/diseases have been demonstrated by several authors (Hardoim et al., 2008; Yuan et al., 2011; Hiruma et al., 2016; Muthukumar et al., 2016), limited studies have provided evidence of fungal spores/conidia growth on infected cadavers exposed to the endophytically colonised host plants (Garrido-Jurado et al., 2017; Gange et al., 2019). Therefore, the reason for the registered mortality might probably be a result of the bioactive secondary metabolites rather than the fungus itself. Also, through the plant-endophyte interaction, some endophytes have been reported to induce the jasmonic acid signalling pathway which mediates plant defense responses against insects, both chewers and phloem feeders, such as whiteflies, reducing their overall fitness (Pappas et al., 2018).

Apart from the effect of endophytes on *T. vaporariorum* development and survival, there was generally higher oviposition on *P. vulgaris* than *S. lycopersicum* even on the control plants. Whiteflies are known to select the most suitable sites for oviposition and the external physical characteristics of the leaf surface, such as hairiness, influence oviposition behaviour (Van Lenteren and Noldus, 1990; Mansaray and Sundufu, 2009). In the present study, the whiteflies preferred *P. vulgaris* to *S. lycopersicum*, a result also supported by other studies where *B. tabaci* and *T. vaporariorum* showed oviposition preference on plants with fewer trichomes (Van Lenteren and Noldus, 1990; Avery et al., 2015). Leaf hairs can act as a physical barrier to oviposition by deterring female whiteflies from resting on the leaf surface and secreting defensive chemicals (Mansaray and Sundufu, 2009; Bar and Shtein, 2019). Notably, *B*. *tabaci* does not oviposit on very hairy *Gossypium hirsutum* varieties (Van Lenteren and Noldus, 1990). However, a contrasting result with the same whitefly species reported oviposition preference on hairy plants, *Glycine max* L. (Mansaray and Sundufu, 2009) and *S. melongena* L. (Shah and Liu, 2013), compared with glabrous ones. Since several other factors such as leaf orientation, colour, texture, and metabolites in the sap all contribute to the oviposition preference by whiteflies (Mansaray and Sundufu, 2009), it can only be postulated that leaf hairiness could be one of the reasons for the observed difference in this study in addition to the endophyte effects, and further research is needed to conclusively determine the cause of the observed variation in the two hosts species.

In conclusion, the use of endophytes is more beneficial, especially against sap-sucking insects such as *T. vaporariorum*. Our results showed the potential of endophytes *H. lixii* F3ST1 and *T. asperellum* M2RT4 as potential biocontrol tools in *S. lycopersicum* and *P. vulgaris* for the management of *T. vaporariorum* due to their suppressive effects on pest survival, oviposition, nymph development time, and adult emergence compared with the control and the other tested endophytes. However, further research needs to be undertaken to understand the mechanisms underlying variations observed concerning endophyte colonisation speed and their effect on oviposition preference by *T. vaporariorum* in the two hosts, *S. lycopersicum* and *P. vulgaris*. Further studies are also warranted to validate the underlined findings under field conditions to integrate these endophytes in the sustainable management of whiteflies in tomato and French bean cropping systems.

## Data Availability Statement

The original contributions presented in the study are included in the article/supplementary material, further inquiries can be directed to the corresponding author.

## Author Contributions

VP, FK, SS, and KA conceived and designed the experiment. VP performed the experiment and analysed the data. VP, KA, AY, SE, SS, and FK wrote the manuscript. All authors have read and agreed to the published version of the manuscript.

## Author Disclaimer

The views expressed in this article do not necessarily reflect the official opinion of the donors.

## Conflict of Interest

The authors declare that the research was conducted in the absence of any commercial or financial relationships that could be construed as a potential conflict of interest.

## Publisher’s Note

All claims expressed in this article are solely those of the authors and do not necessarily represent those of their affiliated organizations, or those of the publisher, the editors and the reviewers. Any product that may be evaluated in this article, or claim that may be made by its manufacturer, is not guaranteed or endorsed by the publisher.
